# Suppression of lung inflammation in an LPS-induced acute lung injury model by the fruit hull of *Gleditsia sinensis*

**DOI:** 10.1186/1472-6882-14-402

**Published:** 2014-10-15

**Authors:** Kyun Ha Kim, Min Jung Kwun, Chang Woo Han, Ki-Tae Ha, Jun-Yong Choi, Myungsoo Joo

**Affiliations:** School of Korean Medicine, Pusan National University, Yangsan, 626-870 Republic of Korea; Korean Medicine Hospital, Pusan National University, Yangsan, 626-870 Republic of Korea; Department of Korean Medical Science, Pusan National University, Yangsan, 626-870 Republic of Korea; Department of Korean Medical Science and Korean Medicine Hospital, Pusan National University, Yangsan, 626-870 Republic of Korea

**Keywords:** *Gleditsia sinensis*, Traditional Asian medicine, Therapeutics, Acute lung inflammation, Nrf2

## Abstract

**Background:**

The fruit hull of *Gleditsia sinensis* (FGS) used in traditional Asian medicine was reported to have a preventive effect on lung inflammation in an acute lung injury (ALI) mouse model. Here, we explored FGS as a possible therapeutics against inflammatory lung diseases including ALI, and examined an underlying mechanism for the effect of FGS.

**Methods:**

The decoction of FGS in water was prepared and fingerprinted. Mice received an intra-tracheal (i.t.) FGS 2 h after an intra-peritoneal (i.p.) injection of lipopolysaccharide (LPS). The effect of FGS on lung inflammation was determined by chest imaging of NF-κB reporter mice, counting inflammatory cells in bronchoalveolar lavage fluid, analyzing lung histology, and performing semi-quantitative RT-PCR analysis of lung tissue. Impact of Nrf2 on FGS effect was assessed by comparing *Nrf2* knockout (KO) and wild type (WT) mice that were treated similarly.

**Results:**

Bioluminescence from the chest of the reporter mice was progressively increased to a peak at 16 h after an i.p. LPS treatment. FGS treatment 2 h after LPS reduced the bioluminescence and the expression of pro-inflammatory cytokine genes in the lung. While suppressing the infiltration of inflammatory cells to the lungs of WT mice, FGS post-treatment failed to reduce lung inflammation in *Nrf2* KO mice. FGS activated Nrf2 and induced Nrf2-dependent gene expression in mouse lung.

**Conclusions:**

FGS post-treatment suppressed lung inflammation in an LPS-induced ALI mouse model, which was mediated at least in part by Nrf2. Our results suggest a therapeutic potential of FGS on inflammatory lung diseases.

**Electronic supplementary material:**

The online version of this article (doi:10.1186/1472-6882-14-402) contains supplementary material, which is available to authorized users.

## Background

Acute lung injury (ALI) is a leading cause of morbidity and mortality in critically ill patients [1]. ALI is most often seen as part of a systemic inflammatory process where the lung manifestations parallel those of other tissues – widespread destruction of the capillary endothelium, extravascation of protein rich fluid and interstitial edema [2]. Gram-negative bacterial infections are the main cause of ALI, and lipopolyssacharide (LPS), a major component of the cell wall of Gram-negative bacteria, is known to be a culprit for the production of reactive oxygen species (ROS) and pro-inflammatory cytokines, resulting in increased infiltration of inflammatory cells [3, 4]. The binding of LPS to TLR4 activates the nuclear factor (NF)-κB so to enhance the production of key pro-inflammatory cytokines including tumor necrosis factor-α (TNF-α), interleukin-1β (IL-1β), IL-6, and monocyte chemotactic protein-1 (MCP-1) [5, 6]. Production of these cytokines contributes to acquisition of neutrophils in the lung, leading to ALI [7]. Given the prevalence and a high mortality of ALI, extensive clinical trials have been conducted for the development of effective treatments for the disease. However, current regimens in clinical settings heavily rely on ventilator and antibiotics [8]. Therefore, ALI remains as an unmet medical need that requires extended and extensive studies for the therapeutics.

Alveolar macrophages are a key responder to LPS and play an important role in regulating lung inflammation [9]. They promote inflammation by producing ROS, along with pro-inflammatory cytokines [10]. Although ROS is a critical molecule to remove pathogens, ROS also cause oxidative stress and inflict damage to the parenchyma of the lung. To cope with these effects by ROS, lung parenchymal cells activate NF-E2-related factor 2 (Nrf2), basic leucine zipper transcription factor, that regulates the expression of various proteins that scavenge ROS, such as NAD(P)H:quinine oxidoreductase 1 (NQO1), heme oxygenase-1 (HO-1), and glutamyl cysteine ligase modulatory (GCLM) and catalytic (GCLC) units [11]. It is well documented that Nrf2 also plays a key role in regulating inflammation in various small animal diseases models [12–14]. Consistent with this, lack of Nrf2 is closely associated with ALI [15]. Thus, Nrf2 can be an excellent therapeutic target for regulation of inflammatory lung diseases.

The fruit hull of *Gleditsia sinensis* LAM (*Leguminosae*) has been used in traditional Asian medicine to treat various respiratory symptoms [16, 17]. However, the diseases targeted by the fruit hull of *G. sinensis* (FGS) have been vaguely defined because it has been prescribed for rather broad symptoms including dyspnea, orthopnea, cough with phlegm and sore throat [18]. In addition, experimental evidence supporting for the therapeutic effects by FGS is scares. A recent study showed that FGS has a preventive effect on lung inflammation in an LPS-induced ALI mouse model and implicated Nrf2 as a possible mechanism for the preventive effect of FGS [19]. This study prompted us to explore the possibility of FGS as a therapeutic candidate against ALI. To do this, we delivered FGS in aerosol to the mouse lung where acute inflammation was elicited by prior LPS treatment, and examined the effect of FGS post-treatment on lung inflammation. Using *Nrf2* KO mice, we determined the role of Nrf2 in the effect of FGS post-treatment on lung inflammation. We found that FGS post-treatment suppressed acute lung inflammation, in which Nrf2 played a role. Thus, our results provide experimental evidence for the suppressive, possibly therapeutic, effect of FGS on respiratory symptoms and a mechanism for the effect.

## Methods

### Preparation of the water extract of *G. sinensis*fruit hull

The fruits of *G. sinensis* were purchased from Kwang-Myoung-Dang herb store (Pusan, Republic of Korea), and authenticated by Professor C.W. Han at the School of Korean Medicine, Pusan National University, Pusan, Republic of Korea. A voucher specimen (number: pnukh001) is kept at the School of Korean Medicine, Pusan National University. The fruit hull of *G. sinensis* (FGS) was separated and manually harvested. A decoction of FGS was obtained by boiling 300 g of FGS in distilled water for 2 hours followed by filtration through 0.45 μm filter. The resultant decoction underwent a freeze-drying process to yield 60 g of powder. Appropriate amount of the powder was dissolved into phosphate buffered saline (PBS) prior to experiment. Fingerprinting the constituents of FGS was performed to ensure the reproducibility of the effect of FGS in the study (Additional file 1: Figure S1).

### Animals

Wild type C57BL/6 and transgenic mice harboring a NF-κB/luciferase reporter construct (C57BL/6 background) were purchased from Jackson laboratory (Bar Harbor, ME 04609, USA). All the mice including *Nrf2* KO (C57BL/6 background) [20] were inbred in a specific pathogen-free (SPF) facility at Pusan National University, Yangsan, Korea (Republic of). Animals were housed in certified, standard laboratory cages, and fed with food and water *ad libitum* prior to experiment. Male mice aged between 7 to 10 weeks old were used for the study.

### Animal model for acute lung injury and FGS administration

All experimental procedures followed the NIH of Korea Guidelines for the Care and Use of Laboratory Animals, and all the experiments were approved by the Institutional Animal Care and Use Committee of Pusan National University (protocol number: PNU-2010-00028). Mice were anesthetized by Zoletil (Virbac, Carros cedex, France), and received a single dose of 10 mg LPS (*Escherichia coli* O55:B5 from Sigma, St. Louis, MO, USA) /kg body weight or sterile saline via intra-peritoneal (i.p.) route. At 2 h after i.p. LPS administration, FGS (150 μg/kg of body weight) in 25 μl of PBS was loaded in a micro-sprayer (Model IA-1C, Penn-Century Inc., USA) and delivered in aerosol to the lung via trachea under visual guidance. The dose of FGS used in the study was determined empirically. Since 3 mg/kg body weight of FGS is normally prescribed for an oral administration to a patient per day in clinic [19], we started with this dose. However, when received 3 mg/kg of intratracheal (i.t.) spraying of FGS, mice were died. Since FGS in aerosol was effective as low as 150 μg/kg, we chose to use 150 μg/kg of FGS for the study. At 24 h after i.t. spraying of FGS, mice were euthanized by CO_2_ gas. The trachea was exposed through midline incision and cannulated with a sterile 24-gauge intravascular catheter. Bilateral bronchoalveolar lavage (BAL) was performed by two consecutive instillations of 1.0 ml of PBS. Total cell numbers in BAL fluid were counted with hemocytometer, and cells in BAL fluid were prepared by a cytospin and stained for the differentiation of macrophages, lymphocytes, or neutrophils by Hemacolor (Merck, Darmstadt, Germany). Three hundred cells in total were counted, and one hundred of the cells in each microscopic field were scored. The mean number of cells per field was reported. For collecting lung tissue, mice were perfused with saline and the whole lung was inflated with fixatives. After paraffin embedding, 5 μm sections were cut and placed on charged slides, and stained with hematoxylin and eosin (H&E) staining method. Three separate H&E-stained sections were evaluated in 100× microscopic magnifications per mouse.

### Bioluminescence

Bioluminescence of the chest of the reporter mice that received i.p LPS with or without subsequent i.t. FGS treatment was measured intermittently for 24 h. Mice were anesthetized with Zoletil (Virbac) before imaging to immobilize them for the duration of the integration time of photon counting (3 min). Mice were shaved over the chest and abdomen before imaging. Luciferin (1 mg/mouse in 100 μl isotonic saline) was administered by i.p. injection and mice were imaged in a supine position with Optix MX3 bioimager and OptiView, a data acquisition program provided by the company (ART Inc, Quebac, Canada). Based on our previously reported results [21], all bioluminescence measurements were done 15 min after i.p. injection of luciferin. For the duration of photon counting, mice were placed inside a light-tight box. Baseline photon counts were obtained before LPS challenge so that each mouse could be used as its own control.

### Western blot analysis

Nuclear proteins from lung tissue were isolated with NE-PER nuclear extraction kit and the manufacture’s protocol (Thermo Scientific, IL, USA). After quantitated by Bradford (Bio-Rad, Hercules, CA, USA), equal amounts of proteins were fractionated by SDS-PAGE and then transferred to PVDF membrane (Bio-Rad). Blots were blocked for at least 1 h with 5% non-fat dry milk prior to incubation with anti-Nrf2 (Santa Cruz Biotechnology, Santa Cruz, CA, USA) or anti-lamin A/C (Santa Cruz Biotechnology) at 4°C overnight. After incubation with secondary antibodies conjugated with HRP for 1 h at room temperature, specific bands of interest were revealed by chemiluminescence (SuperSignal® West Femto, Thermo Scientific).

### Total RNA extraction and semi-quantitative RT-PCR

Total RNA was isolated from right lung homogenates with Ribo-Ex reagent (GeneAll, Seoul, Korea) according to the manufacturer’s instructions. Two micrograms of total RNA was reverse-transcribed by M-MLV reverse transcriptase (Promega, Madison, WI, USA). Target mRNA quantity was determined by using end-point dilution PCR, including three serial 1 to 5 dilutions (1:1, 1:5, 1:25, and 1:125) of RT products for PCR amplification. The level of GAPDH (Glyceraldehyde-3-phosphate dehydrogenase) cDNA from each sample was used to normalize the samples for differences in PCR efficiency. Resultant cDNA was amplified by PCR with a set of specific primers. The forward and the reverse primers for TNF-α were 5′- CTACTCCTCAGAGCCCCCAG - 3′ and 5′- AGGCAACCTGACCACTCTCC - 3′, respectively; and the primers for IL-1β were 5′- - 3′ and 5′- TCGTTGCTTGGTTCTCCTTG - 3′, respectively; the primers for NQO-1 were 5′ – ACTAC GCCATGAAGGAGGCT - 3′ and 5′ – TTCCAGCTTCTTGTGTTCGG - 3′, respectively; the primers for HO-1 were 5′- TGAAG GAGGCCACCAAGGAGG - 3′ and 5′- AGAGGTCAC CCAGGTAGCGGG - 3′, respectively; the primers for GCLC were 5′- CACTGCCAGAACA CAGACCC - 3′ and 5′- ATGGTCTGGCTAGAAGCCT - 3′, respectively; and the primers for GAPDH were 5′- GGAGCCAAAAGGGTCATCAT- 3′ and 5′- GTGATGGCATGGACT GTGGT - 3′, respectively. The reaction conditions were as follows: an initial denaturation at 95°C for 5 min followed by 28 cycles of denaturation for 30 sec at 95°C, annealing for 30 sec at 58°C and extension for 40 sec at 72°C with a final extension for 7 min at 72°C. Amplicons were separated in 1.5% agarose gels. GAPDH was used as internal controls to evaluate relative expressions of TNF-α, IL-1β, GCLC, HO-1, and NQO1. Relative expression of each gene over GAPDH was determined by densitometric analysis software ImageJ (Wayne Rasband, Research Services Branch, National Institute of Mental Health, Bethesda, MD, USA). Reactions were separated in 1.2% agarose gels in 1 × TBE buffer at 100 V for 30 min, stained with SYBR safe DNA gel stain (Invitrogen) and visualized under LED light.

### Statistical analysis

Data are presented as ± SEM (standard error of the mean) of at least three separate experiments. For comparison among groups, one-way analysis of variance (ANOVA) tests with Tukey’s post hoc test was used (with the assistance of InStat, Graphpad Software, Inc., San Diego, CA). *P* values less than 0.05 were considered statistically significant.

## Results and discussion

### FGS post-treatment suppresses lung inflammation in an LPS-induced ALI mouse model

Given the previous report that pretreatment with FGS suppresses lung inflammation in an LPS-induced ALI mouse model, we sought to test whether FGS has a therapeutic potential against inflammatory lung diseases. To this end, we needed to set up a new mouse model because most studies on the effect of a traditional medicine rely on feeding with an herbal medicine for a while prior to the onset of diseases, which address not a therapeutic, but rather preventive, effect of the medicine [22]. In addition, oral feeding may not be an adequate system for studying the effect of an herb on acute inflammatory lung disease such as ALI. To address these, in this study, we delivered FGS directly to the inflamed lung by using a micro-sprayer and examined the effect of FGS on lung inflammation. In our hands, we routinely made 75-80% of the lung received a delivery (data not shown).

**Figure 1:**
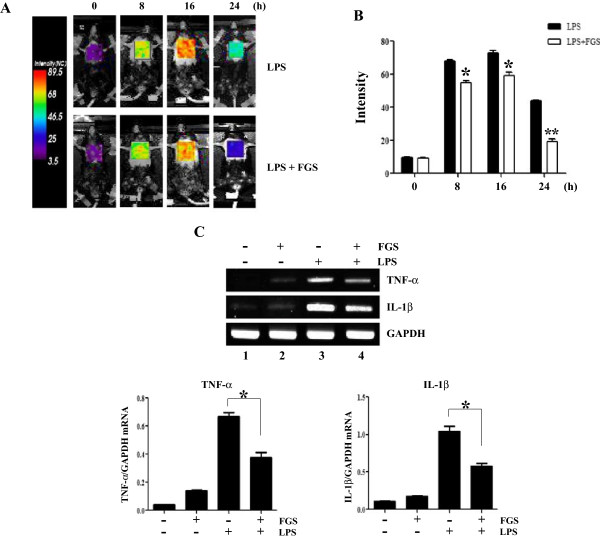
**FGS post-treatment suppresses lung inflammation in an LPS-induced ALI mouse model.** NF-κB reporter mice (n = 5 per group) received an i.p. injection of LPS (10 mg/kg body weight) and subsequently i.t. FGS (150 μg/kg body weight) 2 h after LPS treatment. **(A)** Bioluminescence was measured intermittently for 24 h. Shown are representative bioluminescence images of the chest of 5 reporter mice, which were captured at indicated time points after i.p. LPS injection. The intensity of bioluminescence is shown in color; purple indicates a low level of inflammation and red does a high level of inflammation. **(B)** Bioluminescence was converted to relative photon numbers emitted from the chest of mice. **(C)** At 24 h after LPS treatment, expressions of TNF-α and IL-1β were measured by semi-quantitative RT-PCR of total RNA extracted from the lung of the reporter mice. The intensity of PCR bands of cytokine genes was measured over that of GAPDH by using ImageJ, a densitometric analysis program. Data represent the mean ± SEM of 5 mice. * *P* was less than 0.05, compared with LPS treated group.

To test whether FGS is capable of suppressing lung inflammation, we took transgenic NF-κB reporter mice and measured bioluminescence from the chest of the mice intermittently for 24 h. Since NF-κB regulates the expression of key pro-inflammatory cytokines [23], NF-κB-driven luciferase activity in mouse was measured as a surrogate for inflammatory response. Mice received an i.p. LPS for induction of lung inflammation. At 2 h after LPS treatment, one group received sham and the other did FGS in aerosol by i.t. spraying. As shown in Figure 1A, bioluminescence from the chest was progressively increased to a peak at 16 h after LPS, suggesting that i.p. LPS administration induces lung inflammation (top panels). FGS post-treatment, however, reduced the bioluminescence, which was apparent at 16 h and 24 h after LPS treatment (bottom panels). To quantify the effect of FGS post-treatment on inflammation, we converted chest bioluminescence to relative photon counts. As shown in Figure 1B, photon counts were increased by an i.p. LPS (closed columns), which was reduced to 75 to 50% by FGS post-treatment (open columns). To determine whether FGS post-treatment suppresses the production of pro-inflammatory cytokines, we extracted total RNA from the lungs of the reporter mice, and analyzed it by semi-quantitative RT-PCR for representative pro-inflammatory cytokines, such as TNF-α and IL-1β. As shown in Figure 1C, LPS induced the expression of TNF-α and IL-1β (lane 2), which was decreased by FGS post-treatment (lanes 3 and 4). Together, these results suggest that FGS post-treatment suppresses lung inflammation elicited by an i.p. LPS administration.

### Suppression of lung inflammation by FGS post-treatment is mediated by Nrf2

**Figure 2:**
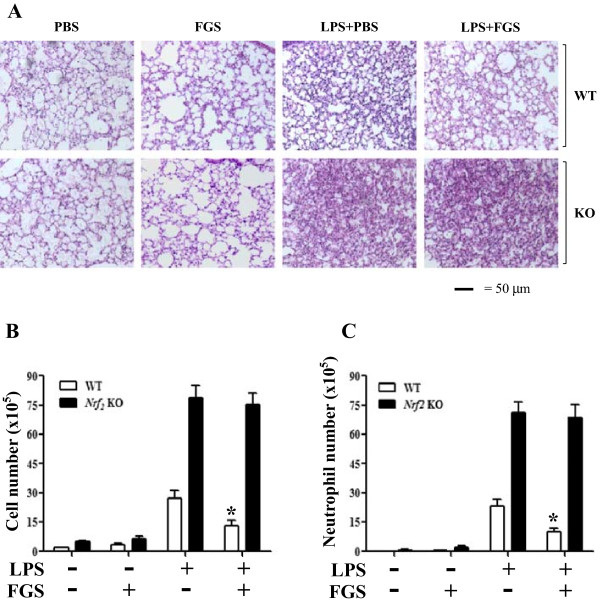
**FGS post-treatment does not reduce lung inflammation in Nrf2 KO mice. (A)** WT and *Nrf2* KO mice (n = 5 per group) received an i.p. PBS or LPS (10 mg/kg body weight). At 2 h after injection, half of each mice group received either an i.t PBS or FGS (150 μg/kg body weight). At 24 h after LPS treatment, lung sections of the mice were stained with H&E for histological examination (magnification × 100). Shown are representatives of at least five different areas of a lung. BAL was performed to differentially count total cells **(B)** and neutrophils **(C)** infiltrated to the lungs of WT and *Nrf2* KO mice. Data represent the mean ± SEM of 5 mice. * *P* was less than 0.05, compared with LPS only. *Nrf2* KO mice show no significant difference between two different treatments.

Given that FGS activates Nrf2 (13), we examined whether Nrf2 is associated with the suppressive effect of FGS post-treatment. WT and *Nrf2* KO mice received an i.p. LPS (10 mg/kg body weight) and 2 h later an i.t. FGS (150 μg/kg body weight). At 24 h after LPS treatment, mice were euthanized for analysis of lung tissue and bronchoalveolar lavage (BAL). Histological analyses of lung sections (Figure 2A) showed that LPS treatment induced cellular infiltration and hyaline change in the lung of WT mice, compared with either PBS or FGS treatment alone (1^st^, 2^nd^ and 3^rd^ top panels). However, FGS post-treatment suppressed lung inflammation in WT mice (4^th^ top panel). By contrast, FGS post-treatment did not significantly suppress lung inflammation in *Nrf2* KO mice treated similarly (3^rd^ and 4^th^ bottom panels).

To determine whether FGS post-treatment suppresses cellular and neutrophil infiltrations to the lung, hallmarks of ALI (2), we analyzed inflammatory cells in BAL fluid. As shown in Figure 2B, LPS treatment increased the number of infiltrated cells in the lung of WT mice (3^rd^ open column), which was significantly reduced by FGS post-treatment (4^th^ open column). Unlike WT mice, however, FGS post-treatment of *Nrf2* KO mice failed to significantly reduce cellular infiltration (3^rd^ and 4^th^ closed columns). Since differential cell counting of BALF showed the prevalence of neutrophils (more than 90%) among infiltrated cells, we also determined the effect of FGS on neutrophil infiltration (Figure 2C). Like Figure 2B, FGS post-treatment significantly suppressed neutrophil infiltration to the lungs of WT mice (3^rd^ and 4^th^ open columns). However, similar treatment did not significantly suppress it in *Nrf2* KO mice (3^rd^ and 4^th^ closed columns). In line with the key role of Nrf2 in suppressing inflammation, *Nrf2* KO mice had about 2.5 times more cellular and neutrophil infiltrations than WT mice (3^rd^ open and closed columns in Figure 2B and C). Although this high degree of infiltration in *Nrf2* KO could explain ineffectiveness of FGS post-treatment of *Nrf2* KO, this possibility remains to be tested. Nevertheless, these results suggest that FGS exerts its suppressive effect on lung inflammation, at least in part, via Nrf2.

### FGS treatment activates Nrf2 and induces Nrf2-dependent gene expression in mouse lung

**Figure 3:**
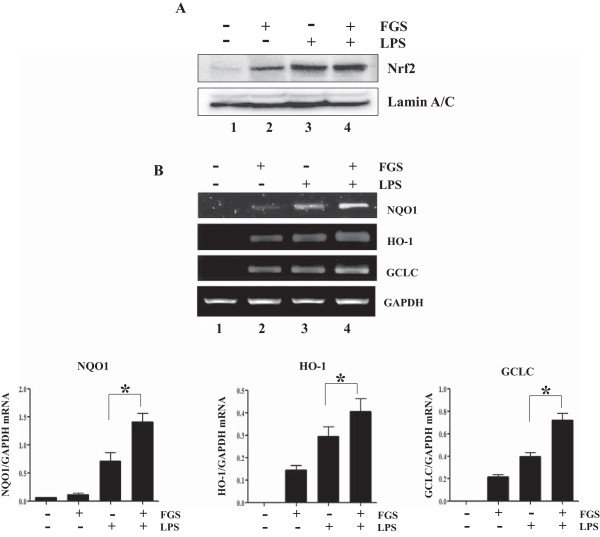
**FGS activates Nrf2 and enhances the expression of Nrf2 dependent genes in mouse lung. (A)** Nuclear proteins were fractionated from the lung tissue, and nuclear Nrf2 was analyzed by western blot. The membrane was stripped and probed again for lamin A/C, a house-keeping protein in the nucleus. **(B)** Expressions of NQO-1, HO-1 and GCLC were measured by semi-quantitative RT-PCR of total RNA extracted from the lung of mice. The intensity of each PCR band was measured over that of GAPDH by ImageJ. Shown are representatives of 5 mouse lungs. Data represent the mean ± SEM of 5 mice. * *P* was less than 0.05.

Since our results indicated that the suppressive effect of FGS is mediated at least in part by Nrf2, we tested whether FGS affects Nrf2 activity in mouse lung. First, since Nrf2 activation is induced by reactive oxygen species (ROS) (10), we sought to exclude the possibility that ROS generated by FGS activate Nrf2. No significant ROS was detected in RAW 264.7 cells treated with FGS (20 μg/ml) (Additional file 1: Figure S2), suggesting that ROS is not involved in Nrf2 activation by FGS. Next, to examine whether FGS activates Nrf2, we took the lungs of WT mice treated with i.p. LPS and subsequent i.t. FGS. At 24 h after LPS, lungs were extracted from mice, and nuclear proteins were fractionated for western blot analysis of nuclear Nrf2, indicative of activated Nrf2 [11]. As shown in Figure 3A, FGS treatment activated Nrf2 in the lung (lane 2). To examine whether FGS activating Nrf2 is related with Nrf2-dependent gene expression, we extracted total RNA from the lung, and performed semi-quantitative RT-PCR for detecting Nrf2-dependent genes. As shown in Figure 3B, FGS treatment enhanced the expression of Nrf2-dependent genes including NQO-1, HO-1 and GCLC (lane 2 and 4) in the lung. Together, these results suggest the possibility that FGS activates Nrf2 to enhance the expression of Nrf2 dependent genes, contributing to the suppressive effect of FGS on lung inflammation.

Given the usage of FGS against respiratory symptoms in traditional Asian medicine and the preventive effect of FGS on lung inflammation reported recently [19], this study was set out to examine the possibility of FGS as therapeutics against inflammatory lung diseases including ALI. Our results show that FGS, administered 2 h after an i.p. injection of LPS, suppressed lung inflammation in an LPS-induced ALI mouse model. While FGS was capable of activating Nrf2 and inducing the expression of Nrf2-dependent genes in the lungs of WT mice, the suppressive effect of FGS on lung inflammation was not detectable in *Nrf2* KO mice. Therefore, our results suggest that the suppressive effect of FGS on lung inflammation is mediated, at least in part, by activating Nrf2. It is unclear, at this moment, how the effect of FGS channels through the activation of Nrf2. Given that FGS is composed of numerous constituents [16, 24–27], it is conceivable that key constituents involved in suppression of inflammation in FGS collectively activate Nrf2. Nevertheless, since Nrf2 plays a key role in protecting various inflammatory diseases, including ALI, asthma, and chronic obstructive pulmonary disease (COPD) [12–14, 20], it is possible that FGS serves as a resource for finding therapeutics against those diseases.

Traditional herbal medicine is usually delivered via oral route. Accordingly, most studies on the therapeutic effect of the medicine have relied on oral administration. In addition, herbal medicine is usually administered several days prior to the onset of disease for a maximum effect. As a result, these studies, including other FGS ones, have addressed rather preventive effects of traditional herbal medicine. In our study, we delivered FGS in aerosol directly to the lungs of the mice after the onset of lung inflammation by prior i.p. LPS administration, and measured the effect of FGS on lung inflammation. Thus, our results contribute to providing experimental evidence that FGS has a therapeutic potential. Our results also suggest that direct delivery of FGS to the lung is effective because single FGS post-treatment was sufficient to suppress lung inflammation. This result is in line with seminal studies that documented the feasibility and efficacy of aerosol intrapulmonary delivery in chemotherapy [28, 29]. Therefore, it is worth considering this intrapulmonary delivery as a new way of treatment in traditional Asian medicine. In this study, we used single protocol to test a potential therapeutic effect of FGS, which is likely insufficient to prove a potential therapeutic effect of FGS on lung inflammation. Clearly, further study will be necessary to consolidate the therapeutic effect of FGS on lung inflammation.

## Conclusion

Although FGS has been prescribed as a traditional herbal medicine against various respiratory symptoms, experimental evidence to support its therapeutic effect has been scares. Our results provide experimental evidence that FGS post-treatment had a suppressive effect on lung inflammation in an LPS-induced ALI mouse model, which was mediated at least in part by Nrf2 activation. Based on our results, we suggest that FGS has a therapeutic potential against inflammatory lung diseases, including ALI.

## Electronic supplementary material

Additional file 1: Figure S1: Fingerprinting of FGS was performed with HPLC (Dionex). FGS run through a column was analyzed by a detector (PDA-100) at UV260 nm and chromelon (Dionex). There are several distinctive and unique peaks in the water extract of FGS. While we don’t know the identity of those peaks, we referred them as key indices for the purpose of quality control. **Figure S2.** Reactive oxygen species (ROS) produced by RAW 264.7 cells treated with FGS or LPS were measured by using the redox sensitive dyes 5-(and-6)-carboxy-2′,7′-dichlorodihydrofluorescein diacetate (carboxy-H2DCFDA, Invitrogen) in conjunction with flow cytometry. No significant ROS was produced by FGS. Shown are representatives of three independent measurements. (PPTX 123 KB)
