# Supplementary material for: Suppression of lung inflammation in an LPS-induced acute lung injury model by the fruit hull of Gleditsia sinensis
Source: BMC Complement Altern Med. 2014 Oct 15;14:402. doi: 10.1186/1472-6882-14-402 (PMC4203922; doi:10.1186/1472-6882-14-402)
Supplement: Supplementary file 1 — Additional file 1: Figure S1: Fingerprinting of FGS was performed with HPLC (Dionex). FGS run through a column was analyzed by a detector (PDA-100) at UV260 nm and chromelon (Dionex). There are several distinctive and unique peaks in the water extract of FGS. While we don’t know the identity of those peaks, we referred them as key indices for the purpose of quality control. Figure S2. Reactive oxygen species (ROS) produced by RAW 264.7 cells treated with FGS or LPS were measured by using the redox sensitive dyes 5-(and-6)-carboxy-2′,7′-dichlorodihydrofluorescein diacetate (carboxy-H2DCFDA, Invitrogen) in conjunction with flow cytometry. No significant ROS was produced by FGS. Shown are representatives of three independent measurements. (PPTX 123 KB) [file 12906_2013_1968_MOESM1_ESM.pptx]

## Slide 1
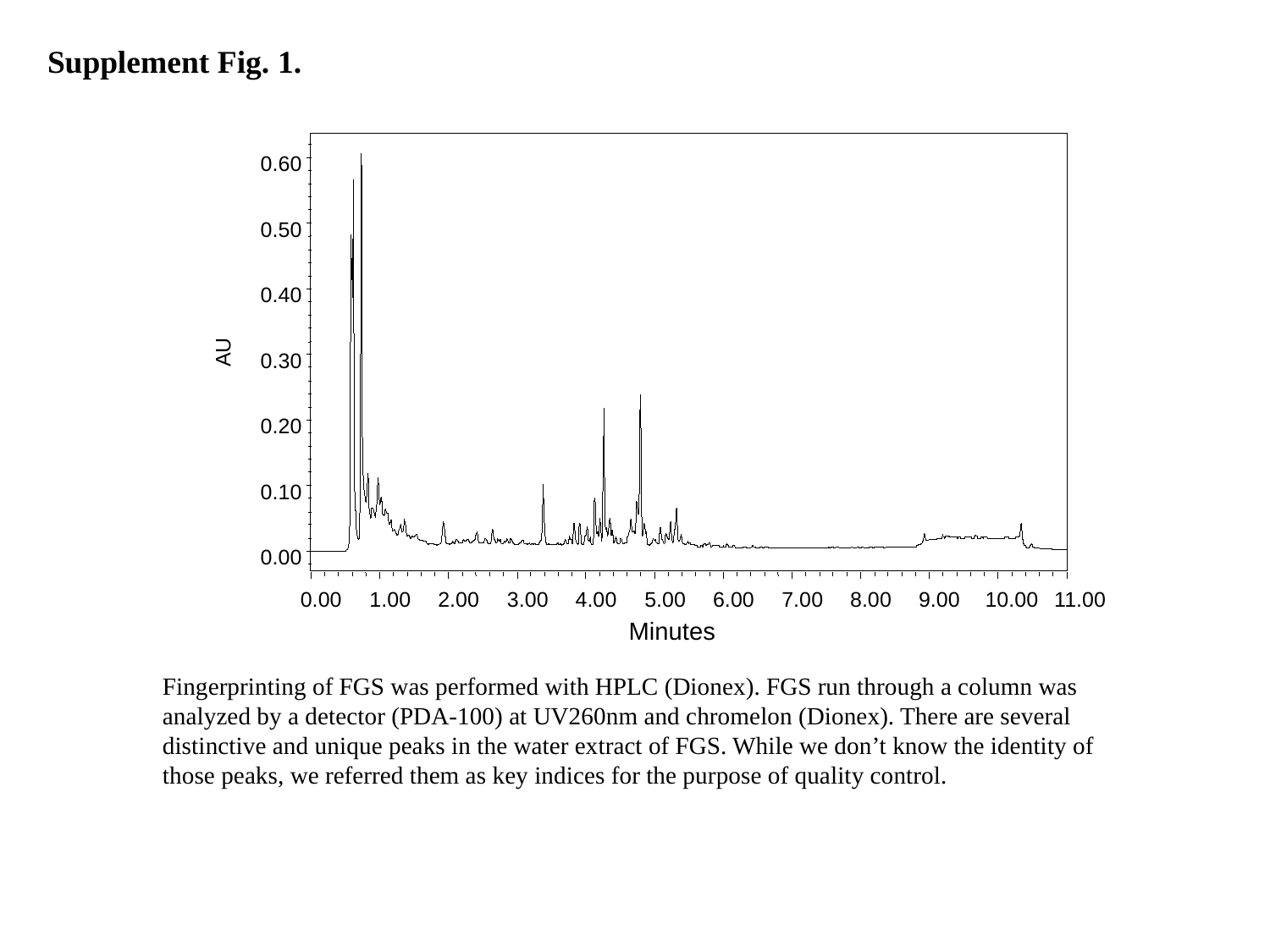

Supplement Fig. 1.
0.60
0.50
0.40
AU
0.30
0.20
0.10
0.00
0.00
1.00
2.00
3.00
4.00
5.00
6.00
7.00
8.00
9.00
10.00
11.00
Minutes
Fingerprinting of FGS was performed with HPLC (Dionex). FGS run through a column was analyzed by a detector (PDA-100) at UV260nm and chromelon (Dionex). There are several distinctive and unique peaks in the water extract of FGS. While we don’t know the identity of those peaks, we referred them as key indices for the purpose of quality control.

## Slide 2
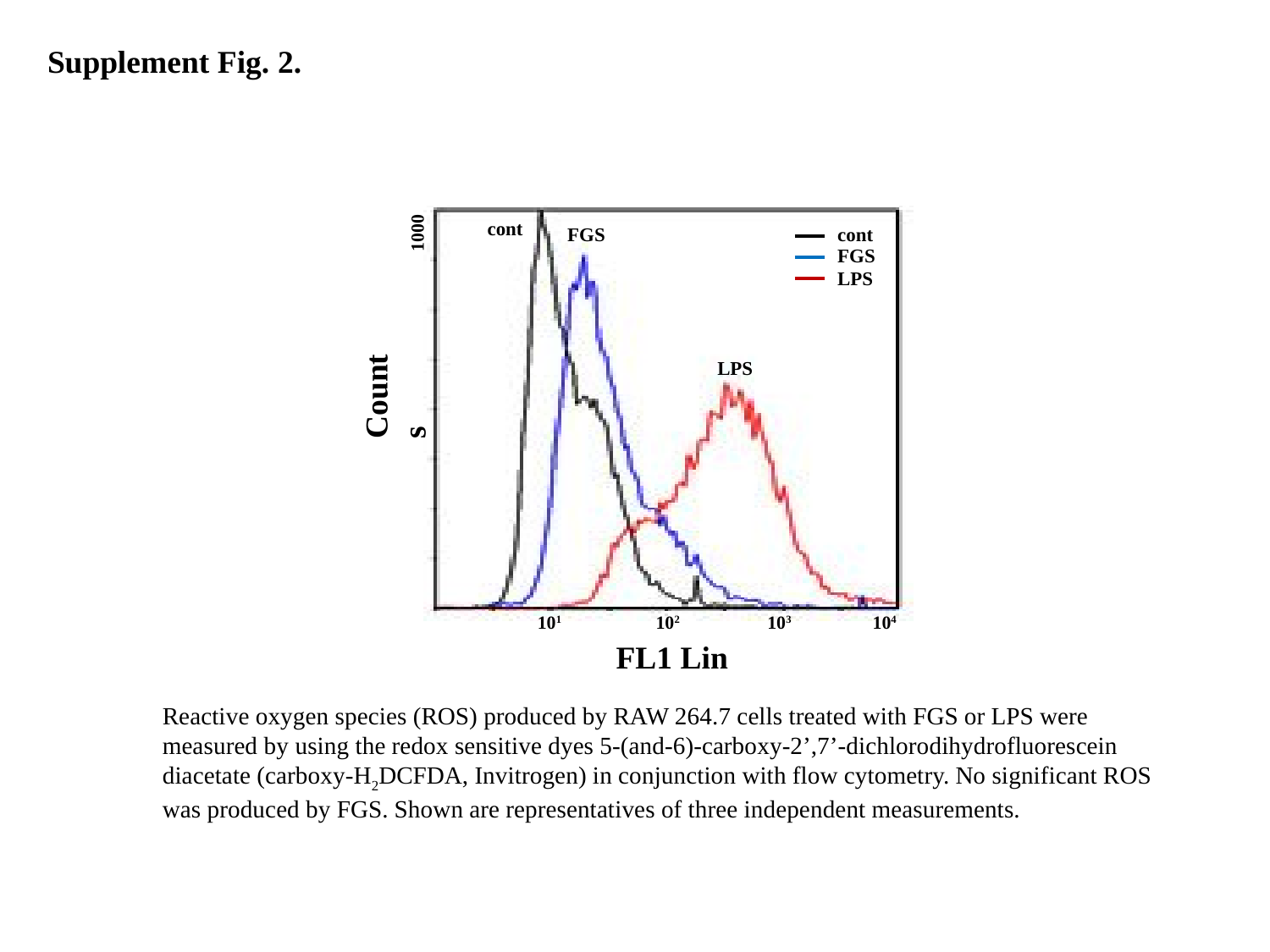

Supplement Fig. 2.
1000
Counts
102
101
103
104
FL1 Lin
cont
FGS
cont
FGS
LPS
LPS
Reactive oxygen species (ROS) produced by RAW 264.7 cells treated with FGS or LPS were measured by using the redox sensitive dyes 5-(and-6)-carboxy-2’,7’-dichlorodihydrofluorescein diacetate (carboxy-H2DCFDA, Invitrogen) in conjunction with flow cytometry. No significant ROS was produced by FGS. Shown are representatives of three independent measurements.
